# The Impact of an Ergonomics Intervention on Psychosocial Factors and Musculoskeletal Symptoms among Thai Hospital Orderlies

**DOI:** 10.3390/ijerph13050464

**Published:** 2016-05-03

**Authors:** Withaya Chanchai, Wanpen Songkham, Pranom Ketsomporn, Punnarat Sappakitchanchai, Wattasit Siriwong, Mark Gregory Robson

**Affiliations:** 1 College of Public Health Sciences, Chulalongkorn University, Bangkok 10330, Thailand; wittt@hotmail.com; 2 Faculty of Nursing, Chiang Mai University, Chiang Mai 50200, Thailand; wsongkhu@hotmail.com; 3 Department of Patient Transfer Service, Faculty of Medicine Siriraj Hospital, Mahidol University, Bangkok 10700, Thailand; siriraj125@gmail.com (P.K.); goodbaby23@hotmail.com (P.S.); 4 School of Environmental and Biological Sciences, Rutgers University, New Brunswick, NJ 08901, USA; robson@aesop.rutgers.edu

**Keywords:** musculoskeletal disorders, physical job demands, psychological perceived job, Thai hospital orderlies

## Abstract

(1) *Background*: Musculoskeletal disorders have a multifactorial etiology that is not only associated with physical risk factors, but also psychosocial risk factors; (2) *Objective*: This study evaluated the effects of an ergonomic intervention on musculoskeletal disorders and psychosocial risk factors; (3) *Material and Methods*: This study took a participatory ergonomic (PE) approach with a randomized controlled trial (RCT) conducted at tertiary care hospitals during July to December 2014. A group of hospital orderlies in Thailand were randomly selected for examination. Fifty orderlies were placed in a case group and another 50 orderlies were placed in the control group. The Nordic Musculoskeletal Disorders Questionnaire (NMQ) and the Copenhagen Psychosocial Questionnaire (COPSOQ) were used for data collection before and after the intervention program; (4) *Results*: The most commonly reported problem among hospital orderlies was found to be lower back symptoms (82%). The study found significant differences in prevalence rates of reported musculoskeletal conditions in the arm, upper back, and lower back regions before and after intervention. Findings showed that psychosocial risk factors were affected by the intervention. COPSOQ psychosocial risk factors were significantly different pre/post intervention. These variables included: work pace, influence at work, meaning of work, predictability, rewards, role conflicts, and social support from supervisors. No other psychosocial risk factors were found to be significant; (5) *Conclusions*: Positive results were observed following the intervention in the work environment, particularly in terms of reducing physical work environment risk factors for musculoskeletal disorders and increasing promotion factors of the psychosocial work environment.

## 1. Introduction

Recently, many studies have shown that musculoskeletal disorders (MSDs) are related to physical and psychological perceived job demands in the work environment [1,2,3,4,5,6]. Risk factors of work related musculoskeletal disorders (WMSDs) are known to include work place activities such as heavy load lifting, repetitive tasks, and awkward working postures [6,7]. Demographic characteristics and psychosocial factors are also known to be important predictive variables [8,9,10,11].

Patient handling activities subject health workers to high biomechanical loads [12,13]. Frequent lifting has been shown to be associated with earlier onset of back injury compared to infrequent lifting, irrespective of hospital orderlies’ occupations [14,15]. Hospital orderlies perform a wide range of job tasks, including prolonged static postures and repetitive tasks, have prolonged periods of exposure to a given task, and undertake a range of physically and psychosocially demanding tasks that have been linked to the development of WMSDs.

Ergonomic training programs are appropriate tools to reduce the prevalence of musculoskeletal disorders caused by psychosocial risk factors [2]. Both passive and active techniques can be implemented during training programs. Passive techniques like lectures are commonly used to share health and safety information. Other passive techniques include communicating information via videos and pamphlets [15]. However, active approaches are preferable. As training techniques move from passive to active, more information is transferred and more changes will be implemented in the workplace [15].

Recently, several studies have been conducted on the impact of ergonomic interventions on psychosocial factors in the work place [16,17,18,19,20,21,22]. These studies have found that ergonomic interventions have improved psychosocial conditions in different working groups. The findings of Haukka *et al.* (2010) [22] did not support the usefulness of participatory ergonomics intervention in changing unsatisfactory psychosocial working conditions. This study was conducted to evaluate effects of ergonomics intervention on musculoskeletal disorders and psychosocial risk factors.

## 2. Methods

### 2.1. Study Setting

This study consisted of a randomized controlled trial (RCT). Two-group pretest-posttest design was conducted from July 2014 to December 2014. The study population consisted of fulltime hospital orderlies employed at a tertiary care hospital setting at the patient transfer service department. Participants had to have been working at this hospital for at least one year. Participants who had any medical history of serious injury, spinal surgery, or severe disability were excluded. Orderlies were part of the 13-unit Patient Transfer Service Department of the facility. Participants from the selected hospital were randomized by employee identification number and allocated into an intervention group (*n* = 50). Participants allocated to the control group (*n* = 50) did not receive ergonomic training. The two groups were studied before and after the intervention. This study was approved by the Ethics Review Committee of Siriraj Institutional Review Board, Faculty of Medicine Siriraj Hospital, Mahidol University, Thailand (COA No. Si296/2014). Participants gave willing consent to participate before any data were collected.

### 2.2. Data Collection

Data collection was conducted through a self-reported, face-to-face questionnaire. The questionnaire gathered information on three categories: participants’ demographic and working data, musculoskeletal problems in different body regions, and perceived job demands. Participants were assigned anonymous identification numbers for future tracking. Participants were given the questionnaire in their workplace. The Nordic Musculoskeletal Questionnaire (NMQ), validated by Kuorinka *et al.* [23], was used to measure the prevalence of MSDs. The psychosocial work environment questionnaire was based on the Copenhagen Psychosocial Questionnaire (COPSOQ) modified by Aust *et al.* [24]. The Thai version of the NMQ was used to examine reported symptoms of MSDs among the study population [25]. The reliability and validity of the Thai version of COPSOQ were examined in a previous study and showed satisfactory psychometric properties [26]. COPSOQ in this study consisted of 57 items in 17 scales. These were:

1. Quantitative demands (3 items)
2. Work pace (1 item)
3. Cognitive demands (4 items)
4. Emotional demands (4 items)
5. Demands for hiding emotions (3 items)
6. Influence at work (4 items)
7. Possibilities for development (4 items)
8. Meaning of work (3 items)
9. Commitment to the workplace (4 items)
10. Predictability (2 items)
11. Rewards (5 items)
12. Role clarity (3 items)
13. Role conflicts (4 items)
14. Quality of leadership (4 items)
15. Social support from supervisor (3 items)
16. Social support from colleagues (3 items)
17. Social community at work (3 items)

Each item was scored based on a five-point scale. There are two kinds of categories set depending on the direction of each question: (1) always, often, sometimes, seldom, never/hardly ever and (2) to a very large extent, to a large extent, somewhat, to a small extent, to a very small extent. Scales were built by summing up the numerical values attached to the response categories of the items. All scales were transformed to a range from 0 to 100: the weights are 0, 25, 50, 75, and 100, to make the scoring on the different scales comparable. Directions of the scores follow the label of the scale; *i.e.*, a high score on the emotional demand scale indicates high emotional demands, a high score on the predictability scale indicates high predictability, and so on. 

The intervention program was developed specifically for the orderlies with a focus on what was relevant to workers in this tertiary care hospital. An important idea guiding the program was that of learning through group conversation and acting within the context of the work environment. Twelve one hour educational training sessions were conducted by the researcher and teams. These sessions provided hospital orderlies with education materials aimed at familiarizing them with the principles of ergonomics, including disorders and workplace conditions, and the objectives of ergonomics intervention. Following training sessions, participants were instructed to remain at their workstations so the trainer could make necessary adjustments. Training sessions provided participants with the necessary skills to assess their work environment and make suggestions for improvements. The trainer provided participants with suggestions and observations, encouraging orderlies to take an active role in adjusting their work improvements achievement. These achievements were categorized into five technical areas of hospital orderlies tasks: (1) Patient care; (2) Safe handling and transferring of patient, medical devices, and equipment; (3) Workstation design; (4) Physical environment; (5) Welfare facilities and administration.

Outcomes were assessed six months after the training sessions took place. Hospital orderlies were asked if they had experienced any MSDs at any time during the previous 12 months. Statistical analyses were performed using SPSS version 16. Groups were compared at baseline using analysis of variance for continuous variables and χ^2^ for categorical variables. A comparison of the groups’ musculoskeletal symptoms before and after the intervention was conducted with a McNemar test. A paired *t*-test was used to compare COPSOQ scores from before and after the intervention. Chi-square tests were used to assess associations between COPSOQ scores and reported musculoskeletal symptoms.

## 3. Results

Table 1 provides descriptive data about the study population including means and standard deviations of age, body mass index, as well as, marital status, educational level, MSDs rate and psychosocial work environment score of the participants in both the case (*n* = 50) and control groups (*n* = 50). As seen, the two groups were similar in terms of demographic variables and no differences were found between case and control individuals.

Table 2 lists musculoskeletal symptoms in the case subjects before and after intervention. The McNemar test indicated significant differences between prevalence rates of reported musculoskeletal symptoms in the arm (*p* = 0.004), upper back (*p* = 0.001), and lower back (*p* = 0.0001) regions before and after intervention. The prevalence rates of problems were significantly lower after intervention.

Table 3 shows musculoskeletal symptoms in the control subjects before and after intervention. McNemar test showed no significant difference in the prevalence rates of musculoskeletal symptoms in all regions before and after intervention.

Table 4 shows the means and standard deviations of scores from the Thai version of the COPSOQ questionnaire for case group individuals before and after intervention. The paired *t*-test showed significant differences in mean scores before and after intervention in: work pace (*p* = 0.002), influence at work (*p* = 0.005), meaning of work (*p* = 0.001), predictability (*p* = 0.001), rewards (*p* = 0.001), role conflicts (*p* = 0.001) and social support from supervisor (*p* = 0.001). No other psychosocial variables were found to be significant. Table 5 also shows that there was no significant difference in any of the scales of the Thai version of the COPSOQ questionnaire before and after intervention in the control group.

## 4. Discussion

The results revealed that prevalence rates of reported symptoms in the arm, upper back, and lower back reduced significantly after the intervention. As expected, results for the lower and upper back showed dramatic differences between the groups. Less change related to wrist pain can be associated with the longer risk exposure times needed for wrist pain to manifest. Interestingly, upper back, lower back and arm symptoms within the case group disappeared after intervention. This may partly be due to the endurance of the erector spinalis and its adaptability to the postural constraints of patient handling and transferring tasks, similar to physical and psychosocial training responses. Ergonomic training should consider the effects of working posture on lower back pain when working in a standing position. Specific ergonomic training focusing on back symptoms meant that this intervention did not affect other regions (*i.e.*, neck, shoulders, wrists-hand, hips/thighs, knee, ankles/feet). This shows that intervention programs have been effective in reducing symptoms in the mentioned regions. This could be attributable to improved orderlies’ awareness of other body regions. No changes were observed before and after intervention in the control group. Our findings are in line with the findings of other studies that reported reductions in MSDs among computer users after attending training [27,28,29,30]. Alireza’s study (2011) found a reduction in back and foot symptoms in oil refinery workers six months after an intervention program took place.

Psychosocial work environment post-intervention scores for positive items (*i.e.*, influence at work, work pace, influence at work, possibilities for development, meaning of work, commitment to the workplace, predictability, role clarity, quality of leadership, and social support from supervisors) among the intervention group were significant. Significance can be attributed to participatory training methods. Improving the work environment through self-initiative may improve workers’ perception of their influence in the workplace. Work improvement activities such as promoting encouragement among coworkers (e.g., increased familiarity between colleagues and supervisors and improved human relations) can improve effectiveness in many ways and can also increase social support at work. However, no significant difference was found among other factors in the intervention group at baseline and post-intervention. The mean score for role conflicts as a negative factor of interpersonal relations showed a significant difference when the intervention group was compared to the control group. Conflicts were considered likely when orderlies perceived their tasks and career roles as highly desirable but mutually exclusive. These results are consistent with previous studies that showed inter-role conflict is likely to increase as job or family demands increase [17]. The training approach not only improved physical aspects, it also led to improvements in the psychosocial work environment. Actively participating in the initiative motivated workers and resulted in improved perceptions of their influences on the work environment. Participatory approaches, such as those aimed to create healthier work environments, can result in increased familiarity between colleagues and supervisors, improved human relations, and can also raise social support at work. None of the other psychosocial variables were found to be significant. This is evidenced through cessation or reduction of both heavy lifting tasks and awkward working postures. These improvements can be attributed to an increased awareness of psychosocial risk factors by hospital orderlies as a result of the interventions put in place, thus reducing job related stress, reducing workplace behavior problems and improving communication between orderlies.

Our findings are similar to the Choobineh, *et al.’s* (2006) study of nurses [31], whereby no significant relationship was found between prevalence rates of musculoskeletal symptoms and psychosocial factors. Previous studies of Thai hospital nurses have also shown that psychosocial factors are not affected by intervention [25]. Kerr *et al.* (2001) [17] indicated that when physical demands were included in a model of musculoskeletal problems, the significance of psychological demands disappeared. Johansson’s (1995) research into psychosocial work factors, physical work load, and associated musculoskeletal symptoms among home care workers concluded that the highest relative risk factors were a combination of a poor psychosocial work environment and high physical workload [17,32]. Alireza *et al.* (2011) [28] noted that some psychological factors, including physical job demands, physical exertion and physical isometric load, were significantly associated with musculoskeletal symptoms in different body regions. Additionally, De Jonge and Kompier (1997) [33] stated that the size of the research population could have important implications for the results of a study [33].

Considering these factors, it is important to point out the limitations of this study. We suggest that, at least, a one-year period is necessary to conduct research of this nature. That amount of time would allow individuals to acquire necessary knowledge from training regarding ergonomics and then apply the knowledge gained to make needed improvements. This is being done in the workplace and people do not want to report problems in case it is reported to management and they feel that they may lose their job. One further limitation, arising from the exclusive use of survey data, is the risk of self-report bias. As both physical and psychosocial factors were assessed through self-reporting, a potential for bias was present. Hospital orderlies may create non-natural links between physical and psychosocial risk factors based on perceived, unfavorable, work environment and health conditions. Future research should explore the relationship between individual tasks and musculoskeletal discomfort. Such analysis may uncover relationships that grouping of high risk tasks might have hidden.

## 5. Conclusions

Following intervention, a reduction in musculoskeletal disorders, particularly in the arms, upper back, and lower back, was observed. Positive results were observed in the work environment, particularly in reducing physical work environment risk factors for musculoskeletal disorders and increasing promotion factors of the psychosocial work environment. However, its effects on health outcomes were questionable and should have been observed over a longer period of time after intervention.

This study demonstrated that training programs that occur in a supportive atmosphere with the full commitment of management were significant in contributing to the success of the intervention.

## Figures and Tables

**Table 1:** Baseline characteristics of 100 hospital orderlies.

Characteristic	Control Group (*n* = 50)	Case Group (*n* = 50)	*p*-Value
Age (years)	34.3 ± 7.3	34.9 ± 9.5	0.725
BMI, kg/m^2^	24.9 ± 4.4	24.8 ± 5.3	0.961
Marital status			
Single	26 (52.0%)	34 (68.0%)	0.105
Married	24 (48.0%)	16 (32.0%)	
Education			
<Bachelor degree	44 (88.0%)	47 (94.0%)	0.229
≥Bachelor degree	6 (12.0%)	3 (6.0%)	
Prevalence rate of MSDs			
Neck	24 (48)	23 (46)	0.843
Shoulders	27 (54)	29 (58)	0.551
Arm	23 (46)	17 (34)	0.225
Upper back	29 (58)	32 (62)	0.543
Wrists-hands	20 (40)	18 (36)	0.684
Lower back	37 (74)	41 (82)	0.339
Hips/thighs	35 (70)	35 (70)	1.000
Knees	25 (50)	30 (60)	0.320
Ankles/feet	27 (54)	21 (42)	0.321
**Psychosocial work environment score, mean** (**SD**)			
**Demand at work**			
Quantitative demands	45.6 (14.4)	46.3 (13.4)	0.781
Work pace	66.0 (30.3)	65.0 (30.3)	0.442
Cognitive demands	57.8 (18.0)	57.1 (18.9)	0.866
Emotional demands	47.1 (19.9)	43.9 (17.5)	0.396
Demands for hiding emotions	55.3 (31.9)	54.3 (13.4)	0.460
**Work organization**			
Influence at work	53.2 (19.2)	49.8 (19.6)	0.375
Possibilities for development	71.3 (12.5)	67.0 (18.0)	0.174
Meaning of work	79.8 (12.6)	78.1 (16.3)	0.555
Commitment to the workplace	60.9 (17.3)	56.9 (14.4)	0.221
**Interpersonal relations at work**			
Predictability	69.5 (17.0)	65.8 (15.3)	0.136
Rewards	68.3 (22.8)	72.8 (11.1)	0.225
Role clarity	67.3 (15.1)	68.2 (14.5)	0.779
Role conflicts	63.9 (14.4)	62.5 (11.8)	0.582
Quality of leadership	66.1 (12.4)	64.7 (12.4)	0.227
Social support from supervisor	54.3 (13.4)	50.5 (14.7)	0.176
Social support from colleagues	51.7 (15.4)	51.0 (15.9)	0.405
Social community at work	60.3 (15.6)	61.3 (12.8)	0.326

**Table 2:** Prevalence rate of reported MSDs in different body regions of case subjects before and after intervention (*n* = 50).

Body Regions	Before Intervention		After Intervention		*p*-Value ^a^
	Yes (%)	No (%)	Yes (%)	No (%)	
Neck	23 (46)	27 (54)	21 (42)	29 (38)	0.500
Shoulders	29 (58)	21 (42)	30 (60)	20 (40)	1.000
Arm	17 (34)	33 (66)	9 (18)	41 (82)	0.004
Upper back	32 (62)	18 (36)	16 (32)	34 (68)	0.001
Wrists-hands	18 (36)	32 (64)	18 (36)	32 (64)	1.000
Lower back	41 (82)	9 (18)	19 (38)	31 (62)	0.001
Hips/thighs	35 (70)	15 (30)	31 (62)	19 (38)	0.125
Knees	30 (60)	20 (40)	27 (54)	23 (46)	0.625
Ankles/feet	21 (42)	29 (58)	16 (32)	34 (68)	0.500

Note: **^a^** McNemar analysis.

**Table 3:** Prevalence rates of reported MSDs in different body regions of control subjects before and after intervention (*n* = 50).

Body Regions	Before Intervention		After Intervention		*p*-Value ^a^
	Yes (%)	No (%)	Yes (%)	No (%)	
Neck	24 (48)	26 (52)	21 (42)	29 (58)	0.250
Shoulders	27 (54)	23 (46)	25 (50)	25 (50)	0.500
Arm	23 (46)	27 (54)	27 (54)	23 (46)	0.250
Upper back	29 (58)	21 (42)	26 (52)	24 (48)	0.500
Wrists-hands	20 (40)	30 (60)	19 (38)	31 (62)	0.301
Lower back	37 (74)	13 (26)	36 (72)	14 (28)	1.000
Hips/thighs	35 (70)	15 (30)	33 (66)	17 (34)	1.000
Knees	25 (50)	25 (50)	21 (42)	29 (58)	0.125
Ankles/feet	27 (54)	23 (46)	26 (52)	24 (48)	0.500

Note: **^a^** McNemar analysis.

**Table 4:** Means and standard deviations of scores of different scales of Thai version of the psychosocial questionnaire in the case before and after the intervention (*n* = 50).

Scales of COPSQ	Mean before Intervention	Mean after Intervention	Mean Difference	Standard Deviation	*t*	*p*-Value ^a^
Quantitative demands (3 items)	46.333	45.833	0.282	1.999	1.769	0.083
Work pace (1 item)	65.000	56.500	2.635	2.635	3.226	0.002 **
Cognitive demands (4 items)	57.125	56.000	0.792	5.602	1.420	0.162
Emotional demands (4 items)	43.925	43.050	0.668	4.726	1.309	0.197
Demands for hiding emotions (3 items)	54.333	52.166	1.185	8.385	1.827	0.074
Influence at work (4 items)	49.750	57.500	2.261	0.534	−2.956	0.005 *
Possibilities for development (4 items)	67.000	67.500	1.007	7.124	−0.496	0.662
Meaning of work (3 items)	78.080	79.913	2.373	3.487	−3.718	0.001 **
Commitment to the workplace (4 items)	56.945	56.570	1.242	8.785	0.302	0.764
Predictability (2 items)	65.820	75.070	2.641	13.141	−3.900	0.001 **
Rewards (5 items)	72.800	75.600	0.687	4.861	−4.073	0.001 **
Role clarity (3 items)	68.167	69.666	1.230	8.697	−1.219	0.229
Role conflicts (4 items)	62.500	69.000	0.874	6.180	−7.436	0.001 **
Quality of leadership (4 items)	67.375	67.000	1.016	7.188	0.369	0.714
Social support from supervisor (3 items)	50.500	57.666	2.462	12.024	−4.214	0.001 **
Social support from colleagues (3 items)	51.000	52.667	1.467	10.378	−1.136	0.262
Social community at work (3 items)	61.333	62.500	1.506	10.649	−0.775	0.442

Note: ^a^ Paired *t*-test. * *p*-value < 0.05, ** *p*-value < 0.01.

**Table 5:** Means and standard deviations of scores of different scales of Thai version of the psychosocial questionnaire in the control before and after the intervention (*n* = 50).

Scales of COPSQ	Mean before Intervention	Mean after Intervention	Mean Difference	Standard Deviation	*t*	*p*-Value ^a^
Quantitative demands (3 items)	45.553	48.053	2.198	15.544	−1.137	0.261
Work pace (1 item)	66.000	70.000	2.065	14.603	−1.937	0.059
Cognitive demands (4 items)	57.750	60.000	2.427	17.163	−0.927	0.358
Emotional demands (4 items)	47.125	48.750	2.641	18.876	−0.165	0.541
Demands for hiding emotions (3 items)	62.833	63.833	3.998	3.998	1.769	0.083
Influence at work (4 items)	63.000	53.625	5.445	38.502	1.772	0.091
Possibilities for development (4 items)	71.250	70.750	1.605	11.325	0.311	0.757
Meaning of work (3 items)	79.833	77.333	1.330	9.411	1.878	0.066
Commitment to the workplace (4 items)	60.857	59.000	1.933	13.672	0.970	0.377
Predictability (2 items)	69.500	65.000	2.251	15.923	1.998	0.051
Rewards (5 items)	68.375	62.500	7.274	5.146	0.808	0.423
Role clarity (3 items)	67.333	64.333	2.239	16.475	1.288	0.204
Role conflicts (4 items)	63.915	62.915	1.000	7.071	1.000	0.322
Quality of leadership (4 items)	66.125	68.250	1.295	9.158	−1.641	0.107
Social support from supervisor (3 items)	54.333	52.333	1.942	13.735	1.303	0.308
Social support from colleagues (3 items)	51.666	51.333	0.751	5.313	0.444	0.659
Social community at work (3 items)	60.333	56.000	2.286	16.169	1.895	0.064

Note: ^a^ Paired *t*-test.

## References

[1] Chanchai W., Songkham W., Ketsomporn P., Sappakitchanchai P., Siriwong W. (2015). Prevalence and Factors Associated with Musculoskeletal Disorders among Thai Hospital Orderlies. Int. J. Occup. Hyg..

[2] Dinora B., Javier C., Aurelio T., Sergio V., Fernando G., Consol S. (2015). Work-related psychosocial risk factors and musculoskeletal disorders in hospital nurses and nursing aides: A systematic review and meta-analysis. Int. J. Nurs. Stud..

[3] Driessen M.T., Proper K.I., Anema J.R., Knol D.L., Bongers P.M., Beek A.J. (2011). The effectiveness of participatory ergonomics to prevent low-back and neck pain results of a cluster randomized controlled trial. Scand. J. Work Environ..

[4] Gilbert-Ouimet M., Brisson C., Vezina M., Trudel L., Bourbonnais R., Masse B., Baril-Gingras G., Dionne C.E. (2011). Intervention study on psychosocial work factors and mental health and musculoskeletal outcomes. Healthc. Pap..

[5] Haukka E., Leino-Arjas P., Ojajärvi A., Takala E.P., Viikari-Juntura E., Riihimäki H. (2011). Mental stress and psychosocial factors at work in relation to multiple-site musculoskeletal pain: A longitudinal study of kitchen workers. Eur. J. Pain.

[6] Vandergrift J.L., Gold J.E., Hanlon A., Punnett L. (2012). Physical and psychosocial ergonomic risk factors for low back pain in automobile manufacturing workers. Occup. Environ. Med..

[7] Bernard B.P. (1997). Musculoskeletal Disorders and Workplace Factors: A Critical Review of Epidemiologic Evidence for Work-Related Musculoskeletal Disorders of the Neck, Upper Extremity, and Low Back.

[8] Haynes S., Williams K. (2008). Impact of seating posture on user comfort and typing performance for people with chronic low back pain. Int. J. Ind. Ergon..

[9] Linton S.J., Kamwendo K. (1989). Risk factors in the psychosocial work environment for neck and shoulder pain in secretaries. J. Occup. Med..

[10] Weiser S., Nordin M., Andersson G.B.J., Pope M.H. (1977). Psychosocial aspects of occupational musculoskeletal disorders. Musculoskeletal Disorders in the Workplace: Principles and Practice.

[11] D’Errico A., Caputo P., Falcone U., Fubini L., Gilardi L., Mamo C., Migliardi A., Quarta D., Coffano E. (2010). Risk factors for upper extremity musculoskeletal symptoms among call center employees. J. Occup. Health.

[12] Marras W.S., Davis K.G., Kirking B.C., Bertsche P.K. (1999). A comprehensive analysis of low-back disorder risk and spinal loading during the transferring and repositioning of patients using different techniques. Ergonomics.

[13] Zhuang Z.Q., Stobbe T.J., Collins J.W., Hsiao H.W., Hobbs G.R. (2000). Psychophysical assessment of assistive devices for transferring patients/residents. Appl. Ergon..

[14] Stobbe T.M., Plummer R.W., Jensen R.C., Attfield M.D. (1988). Incidence of low back injuries among nursing personnel as a function of patient lifting frequency. J. Saf. Res..

[15] Josephson M., Vingard E. (1998). Workplace factors and care seeking for low-back pain among female nursing personnel. Scand. J. Work Environ. Health.

[16] Burke M.J., Sarpy S.A., Smith-Crowe K., Chan-Serafin S., Salvador R.O., Islam G. (2006). Relative effectiveness of worker safety and health training methods. Am. J. Public. Health.

[17] Kerr M.S., Frank J.W., Shannon H.S., Norman R.W., Wells R.P., Neumann W.P., Bombardier C. (2001). Biomechanical and psychological risk factors for low back pain at work. Am. J. Public Health.

[18] Buckle P.W., Devereux J.J. (2002). The nature of work-related neck and upper limb musculoskeletal disorders. Appl. Ergon..

[19] Aarås A., Horgen G., Ro O., Løken E., Mathiasen G., Bjørset H.H., Larsen S., Thoresen M. (2005). The effect of an ergonomic intervention on musculoskeletal, psychosocial and visual strain of VDT data entry work: The Norwegian part of the international study. Int. J. Occup. Saf. Ergon..

[20] Dainoff M.J., Cohen B.G., Dainoff M.H. (2005). The effect of an ergonomic intervention on musculoskeletal, psychosocial, and visual strain of VDT data entry work: The United States part of the international study. Int. J. Occup. Saf. Ergon..

[21] Konarska M., Wolska A., Widerszal-Bazyl M., Bugajska J., Roman-Liu D., Aarås A. (2005). The effect of an ergonomic intervention on musculoskeletal, psychosocial, and visual strain of VDT data entry work: The Polish part of the international study. Int. J. Occup. Saf. Ergon..

[22] Laing A.C., Cole D.C., Theberge N., Wells R.P., Kerr M.S., Frazer M.B. (2007). Effectiveness of a participatory ergonomics intervention in improving communication and psychosocial exposures. Ergonomics.

[23] Haukka E., Pehkonen I., Leino-Arjas P., Viikari-Juntura E., Takala E.P., Malmivaara A., Hopsu L., Mutanen P., Ketola R., Virtanen T. (2010). Effect of a participatory ergonomics intervention on psychosocial factors at work in a randomized controlled trial. Occup. Environ. Med..

[24] Kuorinka I., Jonsson B., Kilbom A., Vinterberg H., Biering-Sorensen F., Andersson G., Jorgensen K. (1987). Standardised Nordic questionnaires for the analysis of musculoskeletal symptoms. Appl. Ergon..

[25] Aust B., Rugulies R., Skakon J., Scherzer T., Jensen C. (2007). Psychosocial work environment of hospital workers: Validation of a comprehensive assessment scale. Int. J. Nurs. Stud..

[26] Songkham W., Siriwong W., Robson M. (2013). Effects of a healthy unit guidance (HUG) program on work environments and health outcomes among nursing personnel. J. Health Res..

[27] Robertson M.M., Huang Y.H., O’Neill M.J., Schleifer L.M. (2008). Flexible workspace design and ergonomics training: Impacts on the psychosocial work environment, musculoskeletal health, and work effectiveness among knowledge workers. Appl. Ergon..

[28] Robertson M., Amick B.C., DeRango K., Rooney T., Bazzani L., Harrist R., Moore A. (2009). The effects of an office ergonomics training and chair intervention on worker knowledge, behavior and musculoskeletal risk. Appl. Ergon..

[29] Gutek B.A., Nakamura C.Y., Nieva V. (1981). The interdependence of work and family roles. J. Occup. Behav..

[30] Mahmud N., Kenny D.T., Md Zein R., Hassan S.N. (2011). Ergonomic training reduces musculoskeletal disorders among office workers: Results from the 6 month follow-up. Malays. J. Med. Sci..

[31] Choobineh A., Motamedzade M., Kazemi M., Moghimbeigi A., Pahlavian A.H. (2011). The impact of ergonomics intervention on psychosocial factors and musculoskeletal symptoms among office workers. Int. J. Ind. Ergon..

[32] Johansson J.A. (1995). Psychosocial work factors, physical work load and associated musculoskeletal symptoms among home care workers. Scand. J. Psychol..

[33] De Jonge J., Kompier M.A.J. (1997). A critical examination of the demand-control support model from a work psychological perspective. Int. J. Stress Manag..

